# Effect of type 2 diabetes on the inducible degrader of LDL receptor

**DOI:** 10.1016/j.jlr.2023.100380

**Published:** 2023-04-23

**Authors:** Sum Lam, David Tak Wai Lui, Sammy Wing Ming Shiu, Ying Wong, Kathryn Choon Beng Tan

**Affiliations:** Department of Medicine, The University of Hong Kong, Hong Kong, China

**Keywords:** diabetes, LDL receptor, monocytes, intracellular lipid, fibroblast growth factor-21

## Abstract

The inducible degrader of LDL receptor (IDOL) acts as a post-transcriptional degrader of the LDL receptor (LDLR). IDOL is functionally active in the liver and in peripheral tissues. We have evaluated IDOL expression in circulating monocytes in subjects with and without type 2 diabetes and determined whether changes in IDOL expression could affect macrophage function like cytokine production in vitro. One hundred forty individuals with type 2 diabetes and 110 healthy control subjects were recruited. Cellular expression of IDOL and LDLR in peripheral blood CD14+ monocytes was measured by flow cytometry. The expression of intracellular IDOL was lower in individuals with diabetes than control (21.3 ± 4.6 mean fluorescence intensity × 1,000 vs. 23.8 ± 6.2, *P* < 0.01), and this was accompanied by an increase in cell surface LDLR (5.2 ± 3.0 mean fluorescence intensity × 1,000 vs. 4.3 ± 1.5, *P* < 0.01), LDL binding, and intracellular lipid (*P* < 0.01). IDOL expression correlated with HbA1c (*r* = −0.38, *P* < 0.01) and serum fibroblast growth factor-21 (FGF21) (*r* = −0.34, *P* < 0.01). Multivariable regression analysis, including age, sex, BMI, smoking, HbA1c, and log(FGF21), showed that HbA1c and FGF21 were significant independent determinants of IDOL expression. IDOL knockdown human monocyte-derived macrophages produced higher concentrations of interleukin 1 beta, interleukin 6, and TNFα than control macrophages upon stimulation with lipopolysaccharide (all *P* < 0.01). In conclusion, the expression of IDOL in CD14+ monocytes was decreased in type 2 diabetes and was associated with glycemia and serum FGF21 concentration.

The LDL receptor (LDLR) mediates the endocytosis of LDL, and the level of expression and activity of the *LDLR* is tightly regulated. Transcriptional regulation of the *LDLR* is mainly by the transcription factors of the sterol regulatory element-binding protein family (1), whereas post-transcriptional mechanism involves the control of the degradation of LDLR. This is mediated by proprotein convertase subtilisin/kexin type 9 (PCSK9) and the inducible degrader of LDL receptor (IDOL) (2, 3). PCSK9 induces the internalization of the LDLR by binding to the extracellular domain of LDLR and is predominantly expressed in the liver. The role of PCSK9 in cholesterol metabolism has been reviewed in detail (4, 5). IDOL, also known as the myosin light chain interacting protein (MYLIP), is an E3 ubiquitin ligase and is ubiquitously expressed (3). Unlike PCSK9, IDOL binds to the intracellular tail of the LDLR through its N-terminal FERM domain. The C-terminal really interesting new gene domain is responsible for its E3 ligase activity. By promoting ubiquitination of the intracellular tail of LDLR, IDOL induces lysosomal degradation of LDLR and inhibits LDL uptake (6). *IDOL* is regulated by the sterol-responsive nuclear receptor liver X receptors (LXRs) (3, 7), and genetic studies have shown that *IDOL* may play a role in LDL metabolism in humans (8, 9, 10).

As both IDOL and PCSK9 are involved in the post-transcriptional regulation of LDLR, it has been suggested that IDOL and PCSK9 might be differentially utilized in a tissue-specific manner (7). Whether IDOL is functionally active in peripheral tissues is under investigation. IDOL has been reported to play a role in a number of tissues, including the liver, intestine, adipose tissue, and the central nervous system (11, 12, 13, 14). Furthermore, IDOL may be one of the main determinants of LDLR expression in macrophages as we and others have shown that PCSK9 is not expressed in macrophages (15, 16, 17). Macrophages play a key role in the pathogenesis of atherosclerosis, and circulating monocyte-derived cells are recruited to the site of atherosclerotic lesion where they differentiate into macrophages and give rise to foam cells (18). Glycemia may potentially influence IDOL expression as glucose concentration has been shown to modulate the expression of LXR-dependent genes like *ABCA1* in macrophages (19). Insulin resistance is associated with dysregulation of lipid metabolism and cellular cholesterol homeostasis, and improvement in insulin resistance caused by weight loss in obese individuals was associated with changes in ABCA1 and IDOL/MYLIP in monocytes (20). Furthermore, Do *et al.* (21) have recently shown that fibroblast growth factor-21 (FGF21) can downregulate MYLIP/IDOL expression in vitro. FGF-21 is mainly produced and secreted by the liver under physiological conditions and is involved in maintaining energy homeostasis. Administering FGF21 has favorable effects on glucose and lipid metabolism in animal studies and reduces body weight (22). Clinical studies have shown that serum levels of FGF21 are elevated in obesity and type 2 diabetes (23). We therefore aimed to determine first, whether type 2 diabetes was associated with changes in IDOL expression and the relationship with glycemia and FGF21. Second, we investigated if changes in IDOL expression affected the function of macrophages like cytokine production in vitro.

## Materials and methods

Subjects with type 2 diabetes were recruited from the diabetes clinics at Queen Mary Hospital. Enrolled subjects must have stable glycemic control with no change in antidiabetic therapy for the preceding 3 months. Key exclusion criteria included type 1 diabetes, statin therapy, history of malignancy or major illness with limited life expectancy, and any hospitalization in the preceding 3 months. Subjects on statins were excluded because statin has been shown to reduce IDOL expression (15). Healthy nondiabetic control subjects were recruited from the community. The study was approved by the Ethics Committee of the University of Hong Kong, and it conforms to the provisions of the Declaration of Helsinki in 1995. Informed consent was obtained from all subjects.

Fasting blood samples were taken for the measurement of glucose, HbA1c, lipids, creatinine, and FGF21, and IDOL and LDLR expression was determined in circulating monocytes. Estimated glomerular filtration rate was calculated using the Chronic Kidney Disease Epidemiology Collaboration equation. Plasma total cholesterol, HDL-C, and triglycerides were determined enzymatically. LDL-C was calculated by the Friedewald equation or measured directly if plasma triglyceride was >4.5 mmol/l. Serum FGF21 was measured using commercially available ELISA (R&D Systems, Minneapolis, MN) according to the manufacturer’s protocol.

**Supplemental Figure S1:**
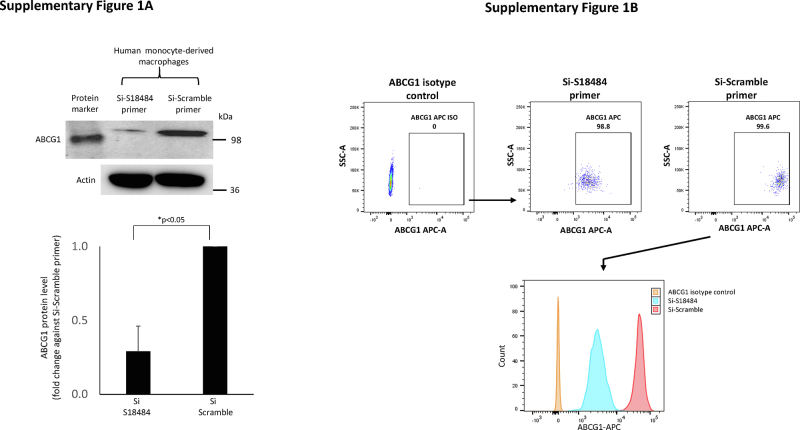
The specificity of anti-ABCG1 antibody was confirmed in ABCG1 knockdown human cells. Human monocyte-derived macrophages from four healthy subjects were transfected with Silencer Select (Si-S18484 or Si-scramble control SiRNA primers, Thermo Fisher Scientific, MA) to knockdown ABCG1 expression. Reduced ABCG1 protein expression was demonstrated by Western blot (A) and by flow cytometry (B).

**Supplemental Figure S2:**
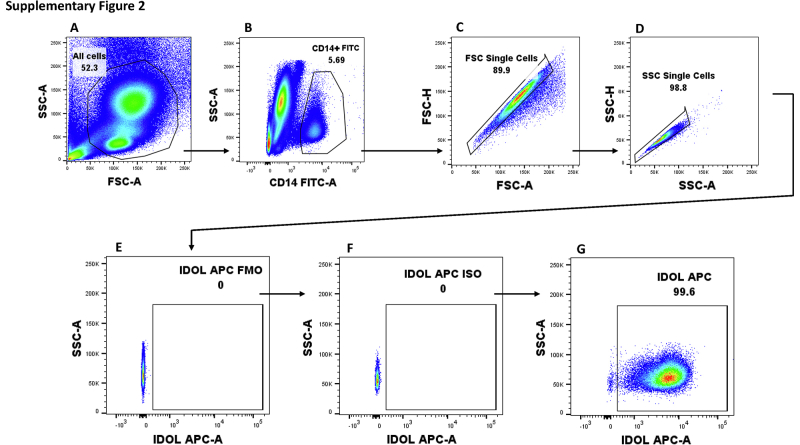
Gating strategy for identification of double positive CD14+/IDOL monocytes using fluorescence minus one (FMO) as gating control. Monocytes were first prestained with CD14+ fluorescein isothiocyanate (FITC) and separated from neutrophil, granulocytes and lymphocytes based on size (forward-scattered light, FSC) (A, B), and granularity/internal complexity (side-scattered light, SSC). An acquisition threshold was set to eliminate platelets, dead cells, and debris. Doublets were further excluded based on FSC area/FSC height (FSC-A/FSC-H) and SSC area/SSC height (SSC-A/SSC-H) (C, D). An allophycocyanin (APC) FMO control was used as gating control (E). Mouse IgG2a Kappa APC-conjugated antibody was used as isotype control to help to differentiate non-specific background signal from positive IDOL-APC specific antibody signal (F). Double positive staining of CD14+/IDOL expression is shown in figure (G). An isotype control was run for each datapoint and the value was subtracted from the measurement.

Cellular expression of IDOL, LDLR, LDL binding, and lipid accumulation in peripheral blood CD14+ monocytes was measured by flow cytometry in all subjects. Cellular expression of IDOL was also further determined by Western blot in a random subgroup of control and diabetic subjects (n = 10). For flow cytometry, antihuman IDOL antibody (catalog no.: SAB1403211; Sigma, St. Louis, MO), anti-ABCG1 (catalog no.: ab204941; Abcam, Cambridge, UK), and anti-LDLR antibody (catalog no.: ab204941, Abcam, Cambridge, UK) were first conjugated with allophycocyanin (APC) using Alexa Fluor™ 647 Antibody Labeling Kits (catalog no.: A02186; Thermo Fisher Scientific, MA) according to the manufacturer’s protocol. Fifty microliters of anticoagulated whole blood freshly collected from participants was incubated for 10 min at room temperature with 2 ml of Pharmalysze solution (BD Biosciences, San Jose, CA). To differentiate monocytes from other leukocytes, anti-human CD14+ IgG conjugated with FITC (catalog no.: ab28061; Abcam, Cambridge, UK) was first added and incubated on ice for 30 min. To measure surface LDLR, anti-LDLR antibody as the primary antibody was incubated with CD14-positive labeled monocytes for 30 min at 4°C. For intracellular IDOL and ABCG1 detection, CD14-positive labeled monocytes were permeabilized and fixed using Fixation/Permeabilization Solution Kit (BD Biosciences) prior to immunostaining with APC-conjugated anti-human IDOL antibody and anti-ABCG1, respectively. The specificity of anti-ABCG1 antibody had been confirmed in ABCG1 knockdown human cells by Western blot and flow cytometry (supplemental Fig. S1). To quantify the amount of surface LDL binding and intracellular lipid accumulation, APC fluorochrome-conjugated LDL (10 μg/ml) and Nile-Red (10 μg/ml) (Invitrogen, Waltham, MA) were used directly on the stained monocytes, respectively. The samples were finally analyzed with BD LSR Fortessa Analyzer (BD Biosciences). A fixed number of 10,000 monocytes were analyzed for each sample. Mean fluorescence intensity (MFI) was analyzed with FAScan (BD Biosciences), and the results were presented as the magnitude increase of MFI (MFI units of tested antibody staining minus MFI units of control antibody staining). Data were analyzed using FlowJo software, version X.0.7 (Tree Star, Inc). Gating strategy for flow cytometry is shown in supplemental Fig. S2. Gates were drawn according to respective fluorescence minus one controls. CD14+ monocytes in peripheral blood mononuclear cells isolated from a healthy individual were used as an internal control, and an aliquot of stored blood mononuclear cells was included in each run. The intra and inter coefficients of variation were 5.49% and 9.71%, respectively.

**Supplemental Figure S3:**
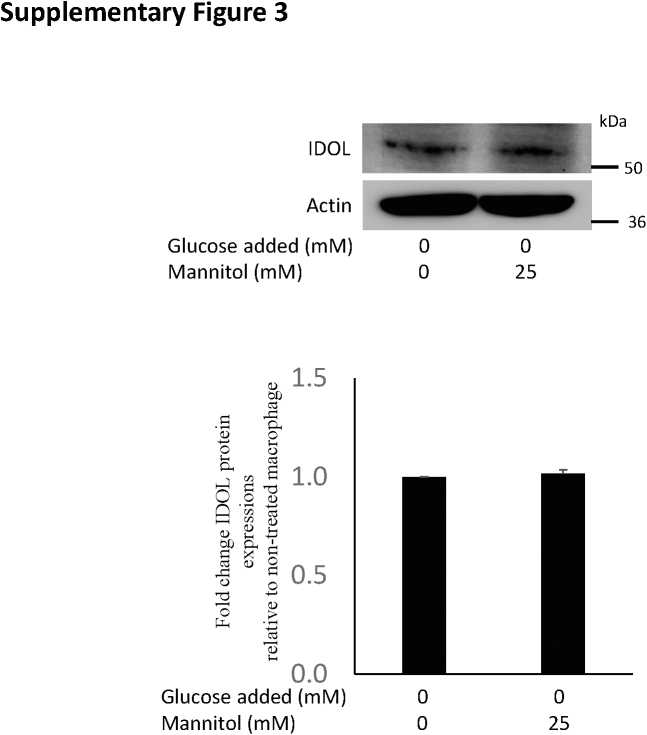
To control for osmotic effect on IDOL expression, human monocyte-derived macrophages were treated for 24 h with or without 25 mM mannitol added to the culture medium. Data represent the mean ± SD of four experiments.

To investigate whether downregulation of IDOL in diabetes was mediated by hyperglycemia and/or FGF21, human monocyte-derived macrophages were incubated with glucose or FGF21. Peripheral blood mononuclear cells were first isolated from normal control subjects using Ficoll–Paque plus density gradient separation (St. Louis) and were allowed to differentiate into monocyte-derived macrophages using RPMI 1640 medium with 10% autologous serum and 5 mM glucose. Macrophages were then either treated with increasing concentrations of FGF21 (10, 20, and 50 ng/ml), or further added glucose (0, 5, 12.5, and 25 mM), or mannitol (25 mM) as an osmotic control for 24 h (supplemental Fig. S3). Cells were lyzed, and mRNA expression of IDOL was determined by TaqMan gene expression assay (Hs00982312_m1; Applied Biosystems, Grand Island, NY). Samples were run in triplicates, and predesigned primers and probes for GAPDH (Hs99999905_m1; Applied Biosystems) were amplified simultaneously in a separate tube as a house-keeping reference gene to the IDOL target. Protein expressions of IDOL were also determined by Western blot analysis.

In vitro RNA silencing experiments were performed to determine whether changes in IDOL expression affected intracellular lipid accumulation and the function of macrophages. *IDOL* gene knockdown in human monocyte-derived macrophages was performed using silencing IDOL RNA gene techniques (Origene, Rockville, MD). Macrophages were incubated with 10 μg/ml LDL for 6 h and then stained with Oil Red O and hematoxylin to visualize lipid accumulation. Images were acquired using the 40× objective on a microscope (model BX51; Olympus) equipped with a digital camera (model DP70; Olympus) using the ImagePro Plus software program (Media Cybernetics, Inc.). Intracellular cholesterol was also quantified. Hexane/isopropanol was used to extract intracellular lipids, and cellular cholesterol levels were then determined by commercially available kits (Wako Chemical, VA). Total cellular protein levels were measured by Lowry method. Results were expressed in micrograms of cholesterol per milligrams of cellular protein.

To determine the effect of IDOL knockdown on cytokine production, cells were treated with or without lipopolysaccharide (LPS) (100 ng/ml, 48 h), and cytokines were measured in the supernatant. TNFα, interleukin 6, and interleukin 1 beta were assayed according to the protocol provided (R&D Systems). Further experiments were performed to investigate whether upregulation of cytokine production induced by LPS stimulation in IDOL knockdown macrophages was due to increased activation of the NF-kB proinflammatory pathway. To evaluate the phosphorylation status of LPS-treated macrophages, phospho-specific antibodies (1:1,000 dilution; Cell Signaling) of phospho-NF-kB p-65 was performed by Western blot analysis. Total NF-kB p-65 was used as normalization control to phospho-NF-kB p-65. Beta-actin was employed for protein loading control. All experiments were repeated four times, and results were expressed as mean ± SD.

Results were expressed as mean and SD or as median and interquartile range if the distribution of the data was not normally distributed. Skewed data were logarithmically transformed before analyses were made. Student's *t*-test and one-way ANOVA were used to compare variables between two and multiple groups, respectively. Pearson’s correlation coefficient and multiple linear regression analysis were used to test the relationships between variables.

## Results

One hundred forty individuals with type 2 diabetes and 110 healthy control subjects were recruited. The clinical characteristics are shown in Table 1. None of the subjects had history of cardiovascular disease as we have excluded subjects on statin therapy in our study. As expected, subjects with diabetes had higher BMI, fasting glucose, and HbA1c than control. Serum FGF21 level was significantly elevated in subjects with diabetes but was no longer significant after adjusting for age, sex, and BMI.

**Table 1:** Clinical Characteristics of control and subjects with type 2 diabetes

Clinical and biochemical parameters	Control	Type 2 diabetes mellitus
N	110	140
Age (years)	52.6 ± 12.3	55.7 ± 8.7∗
Male/female (%)	44/56	48/52
Duration of diabetes (years)	0	11.7 ± 8.5
BMI (kg/m^2^)	23.7 ± 3.7	27.5 ± 5.3∗∗
Smoker (%)	9.1	10.8
Hypertension (%)	0	70.7
Systolic blood pressure (mm Hg)	124 ± 15	135 ± 18∗∗
Diastolic blood pressure (mm Hg)	76 ± 10	78 ± 9
Fasting glucose (mmol/l)	5.1 ± 0.5	7.6 ± 2.8∗∗
HbA1c (%)	5.6 ± 0.4	7.9 ± 1.8∗∗
Estimated glomerular filtration rate (ml/min/1.73 m^2^)	88 (79–95)	81 (64–93)
Total cholesterol (mmol/l)	5.28 ± 0.84	5.42 ± 0.94
Triglyceride (mmol/l)	1.02 (0.72–1.52)	1.40 (1.03–2.18)∗∗
LDL-C (mmol/l)	3.18 ± 0.82	3.22 ± 0.79
HDL-C (mmol/l)	1.40 ± 0.40	1.22 ± 0.41∗∗
FGF21 (pg/ml)	89.6 (50.7–153.8)	126.4 (71.2–184.5)∗∗

Data are expressed as mean ± SD or median (interquartile range).

∗*P* < 0.05 and ∗∗*P* < 0.01 compared with control.

**Fig. 1:**
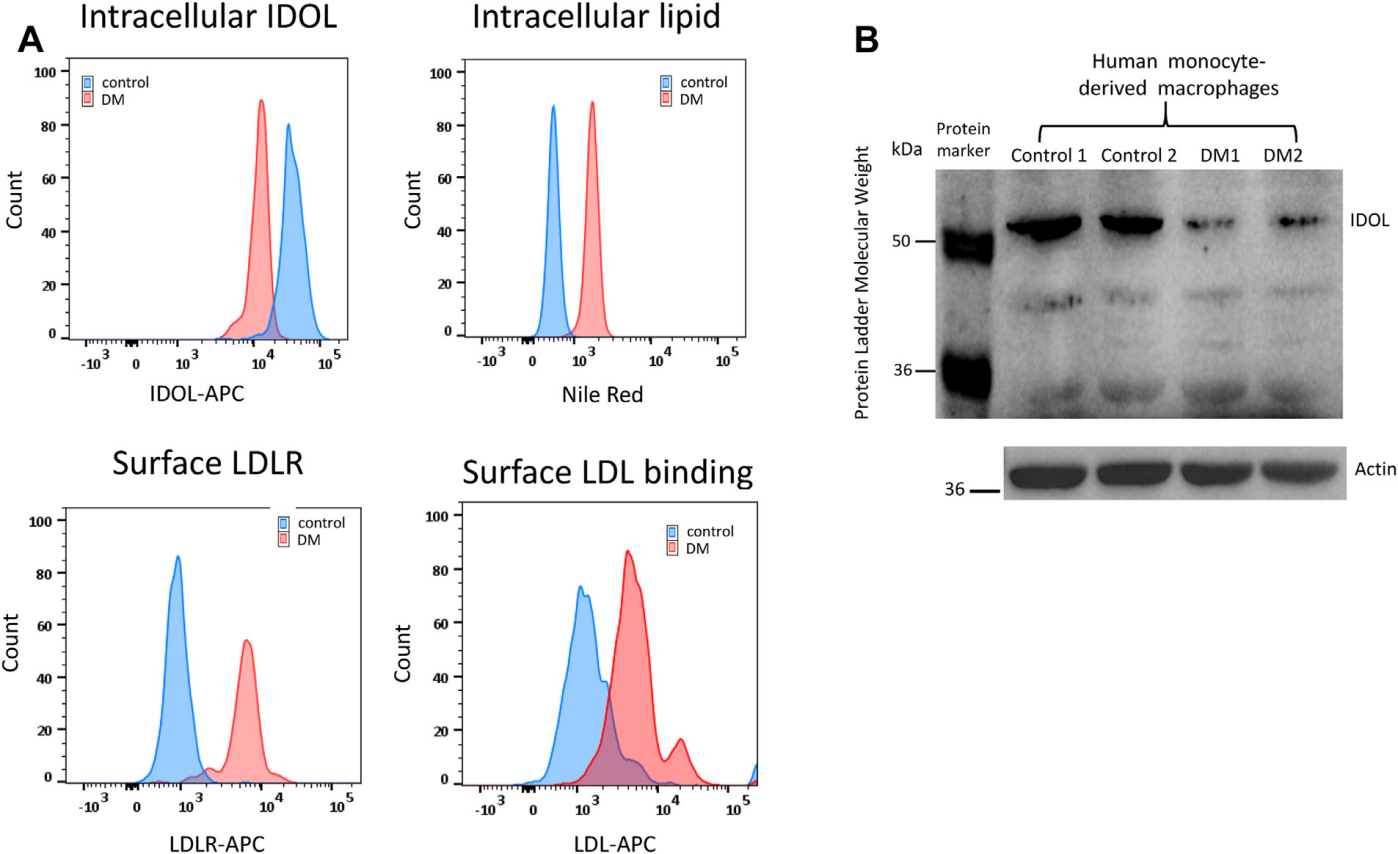
Representative figures of all flow cytometry measurements (A) and Western blots of IDOL (B) in type 2 diabetes mellitus and control.

The data on IDOL and LDLR expression in CD14+ monocytes are shown in Table 2, and representative figures of all flow cytometry measurements are shown in Fig. 1A. The expression of intracellular IDOL was significantly lower in individuals with diabetes than control, and this was confirmed by Western blot (Fig. 1B). The reduction in IDOL expression was accompanied by an increase in the expression of cell surface LDLR and in the binding of LDL as well as an increase in intracellular lipid accumulation. The differences in all these parameters between subjects with diabetes and control remained significant even after adjusting for age, sex, and BMI (*P* < 0.01). The relationship between IDOL expression and cell surface LDLR expression, LDL binding, and intracellular lipid accumulation is shown in Table 3. IDOL expression inversely correlated with LDLR expression, LDL binding, and intracellular lipid content.

**Table 2:** Expression of IDOL and LDLR in CD14+ monocytes in control and subjects with type 2 diabetes

Monocyte IDOL and LDLR	Control	Type 2 diabetes mellitus
Intracellular IDOL (MFI × 1,000)	23.8 ± 6.2	21.3 ± 4.6∗∗
Surface LDLR (MFI × 1,000)	4.3 ± 1.5	5.2 ± 3.0∗∗
LDL binding (MFI × 1,000)	4.0 ± 1.4	4.9 ± 2.0∗∗
Intracellular lipid (MFI × 1,000)	7.7 ± 2.7	10.9 ± 4.7∗∗

Data are expressed as mean ± SD.

∗∗*P* < 0.01 compared with control.

**Table 3:** Correlations between IDOL expression and LDLR, LDL binding, and intracellular lipid accumulation in CD14+ monocytes

Parameters	IDOL	LDLR	LDL binding
Type 2 diabetes mellitus			
LDLR	−0.61∗∗		
LDL binding	−0.47∗∗	0.38∗∗	
Intracellular lipid	−0.33∗∗	0.36∗∗	0.25∗∗
Control			
LDLR	−0.43∗∗		
LDL binding	−0.33∗∗	0.32∗∗	
Intracellular lipid	−0.39∗∗	0.45∗∗	0.29∗∗

Data are Pearson’s correlation coefficients.

∗∗*P* < 0.01.

**Supplemental Figure S4:**
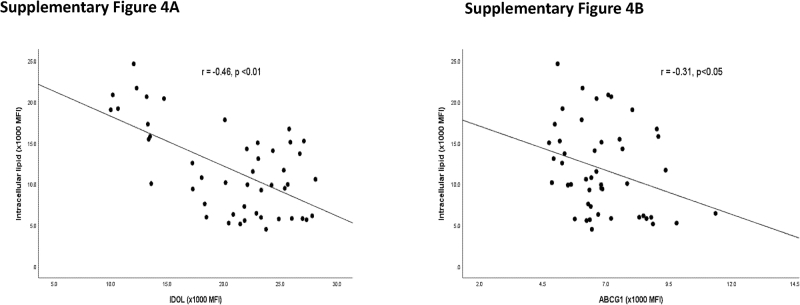
Pearson's correlation analysis of the association between intracellular lipid accumulation and IDOL expression (A) and ABCG1 expression (B) inCD14+ monocytesin type 2 DM.

Mauldin *et al.* (24) have shown that monocytes from type 2 diabetic patients show increased lipid accumulation because of a decrease in ABCG1 expression. We have also previously reported that the expression of ABCG1 was significantly reduced in monocytes in patients with type 2 diabetes and was associated with impaired cholesterol efflux, whereas no changes were seen in ABCA1 and SR-B1 compared with controls (25). Hence, another 47 patients with type 2 diabetes were recruited, and IDOL and ABCG1 expression was determined to evaluate the contribution of IDOL and ABCG1 to intracellular lipid accumulation. Both the expression of IDOL (*r* = −0.46, *P* < 0.01) and ABCG1 (*r* = −0.31, *P* < 0.05) correlated with intracellular lipid accumulation (supplemental Fig. S4A, B). Forward stepwise linear regression analysis showed that both IDOL and ABCG1 contributed to intracellular lipid accumulation, accounting for 26% and 15% of the variation in intracellular lipid respectively (adjusted *R*^2^ of the model = 0.41, *P* < 0.01).

**Fig. 2:**
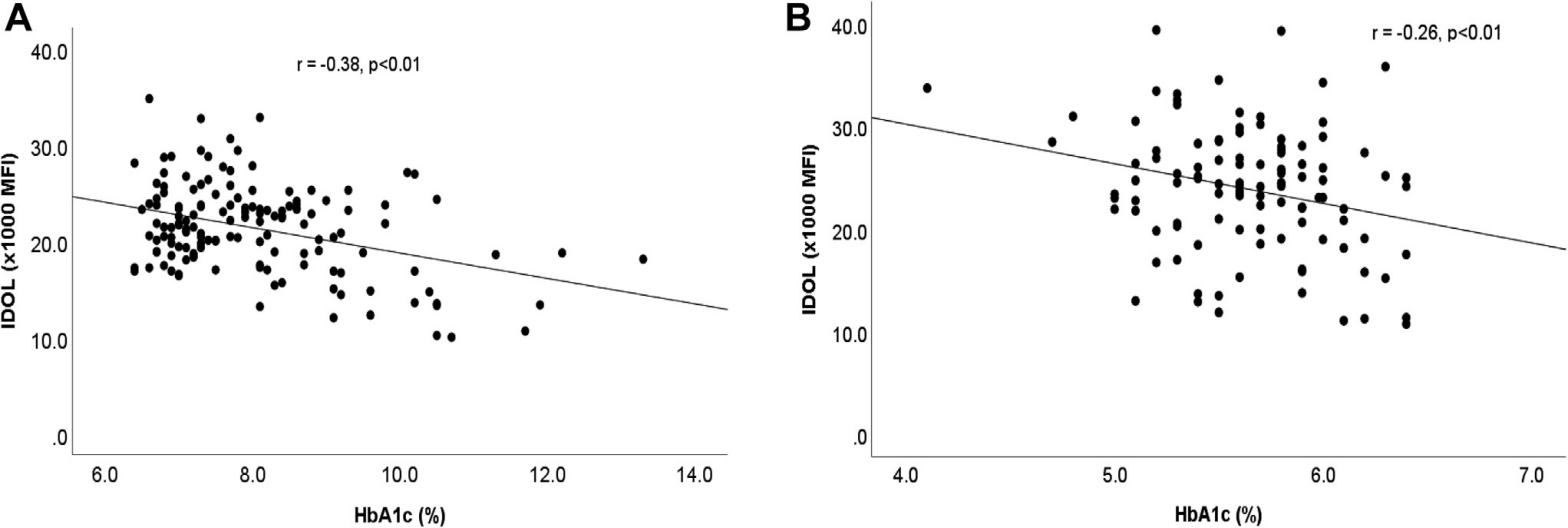
Pearson's correlation analysis of the association between HbA1c and IDOL expression in CD14+ monocytes in type 2 diabetes mellitus (A) and control (B).

**Fig. 3:**
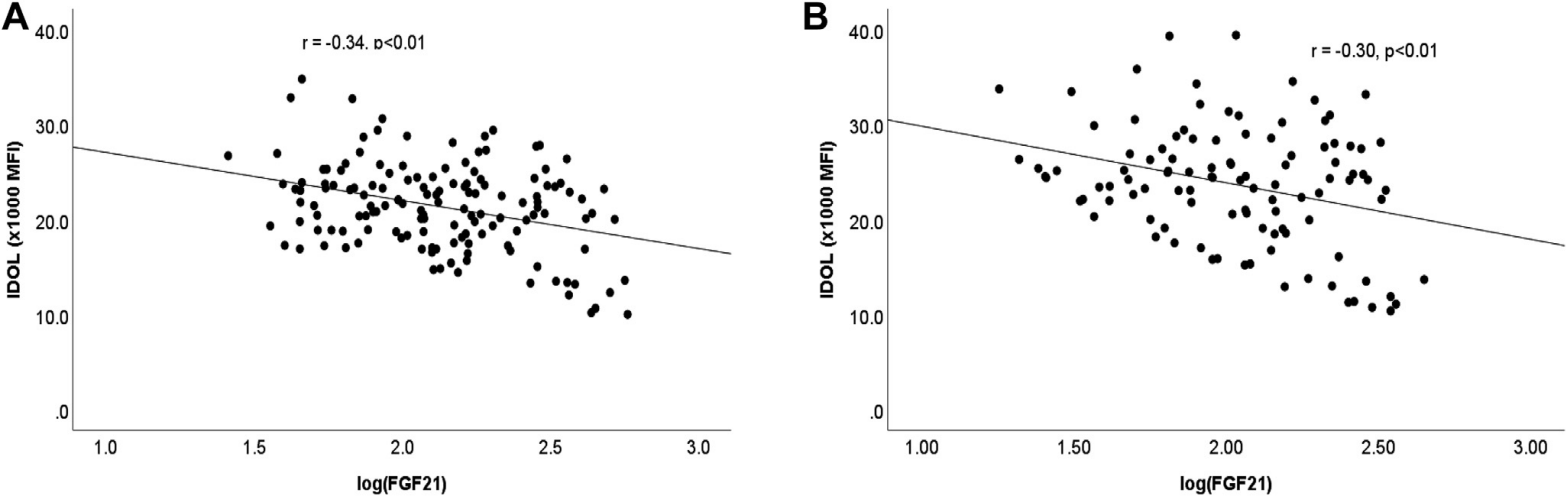
Pearson's correlation analysis of the association between FGF21 and IDOL expression in CD14+ monocytes in type 2 diabetes mellitus (A) and control (B).

**Fig. 4:**
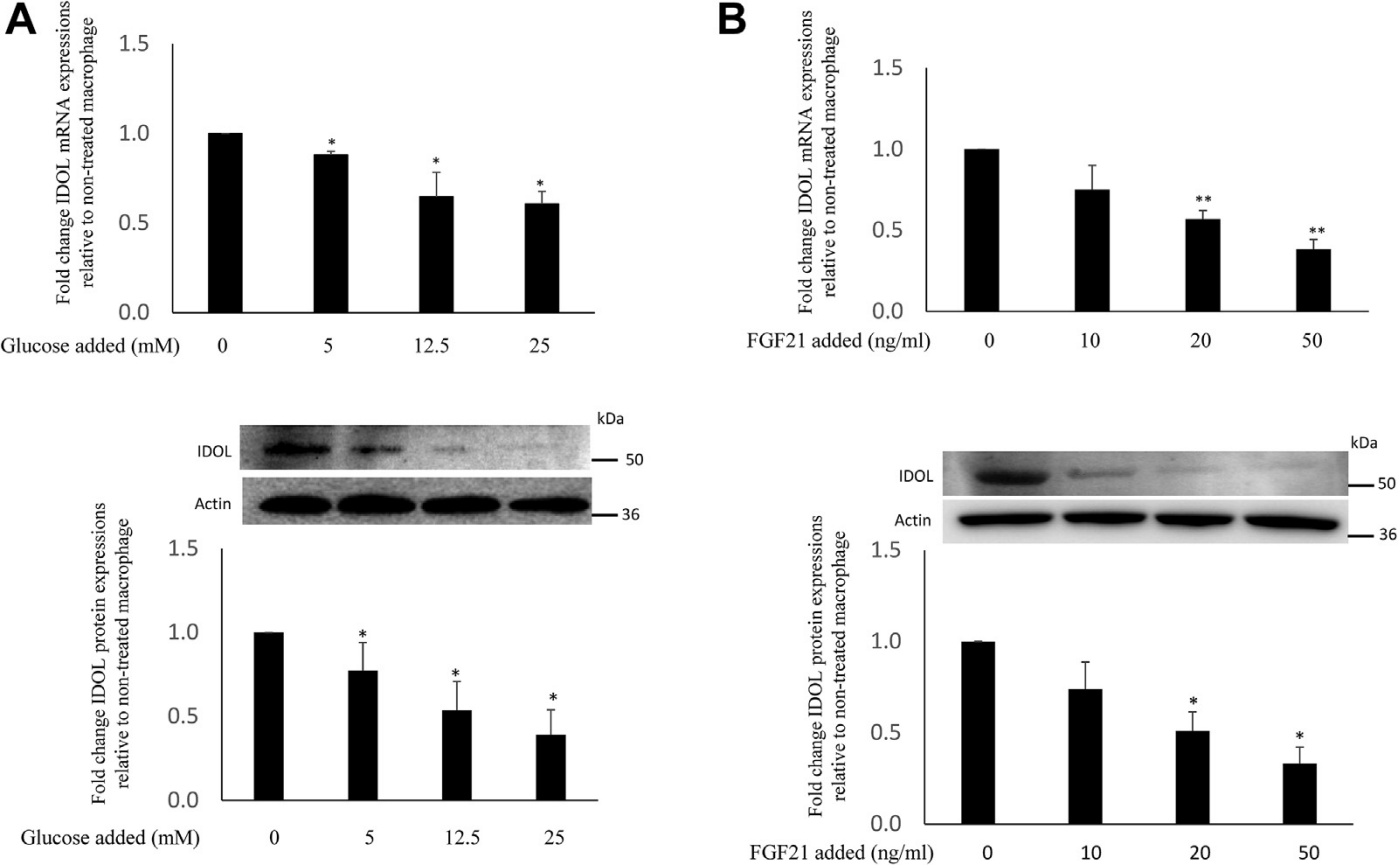
Effect of glucose (A) and FGF21 (B) on IDOL mRNA and protein expression in human monocyte-derived macrophages. Peripheral blood mononuclear cells were allowed to differentiate into monocyte-derived macrophages using RPMI 1640 medium with 10% autologous serum and 5 mM glucose. Cells were then treated for 24 h with different concentrations of added glucose and FGF21, respectively, and IDOL was determined. Data represent the mean ± SD of four experiments. ∗*P* < 0.05; ∗∗*P* < 0.01 for treated versus control cells. The statistical significance of differences was determined using ANOVA followed by Bonferroni’s comparison.

To investigate the potential mechanisms of diabetes-mediated downregulation of IDOL, univariate analysis showed that there were negative correlations between HbA1c and FGF21 with IDOL expression both in individuals with and without diabetes (Figs. 2 and 3, respectively). IDOL expression did not correlate with age, BMI, plasma lipid levels, or estimated glomerular filtration rate in individuals with diabetes or in control. Since the associations between HbA1c, FGF21, and IDOL were similar in subjects with and without diabetes, the two groups were combined in subsequent analysis. Forward stepwise linear regression analysis, including age, sex, BMI, smoking, HbA1c, and log(FGF21), showed that both HbA1c and FGF21 were significant independent determinants of IDOL expression, accounting for 15% and 6% of variation in IDOL expression. There was no significant interaction between HbA1c and FGF21. In vitro experiments were therefore carried out to determine the effects of glucose and FGF21 on IDOL expression. Incubation of human monocyte-derived macrophages with glucose showed a reduction in IDOL expression (Fig. 4A). FGF21 also downregulated IDOL expression in macrophages (Fig. 4B), consistent with the findings of Do *et al.* (21).

**Fig. 5:**
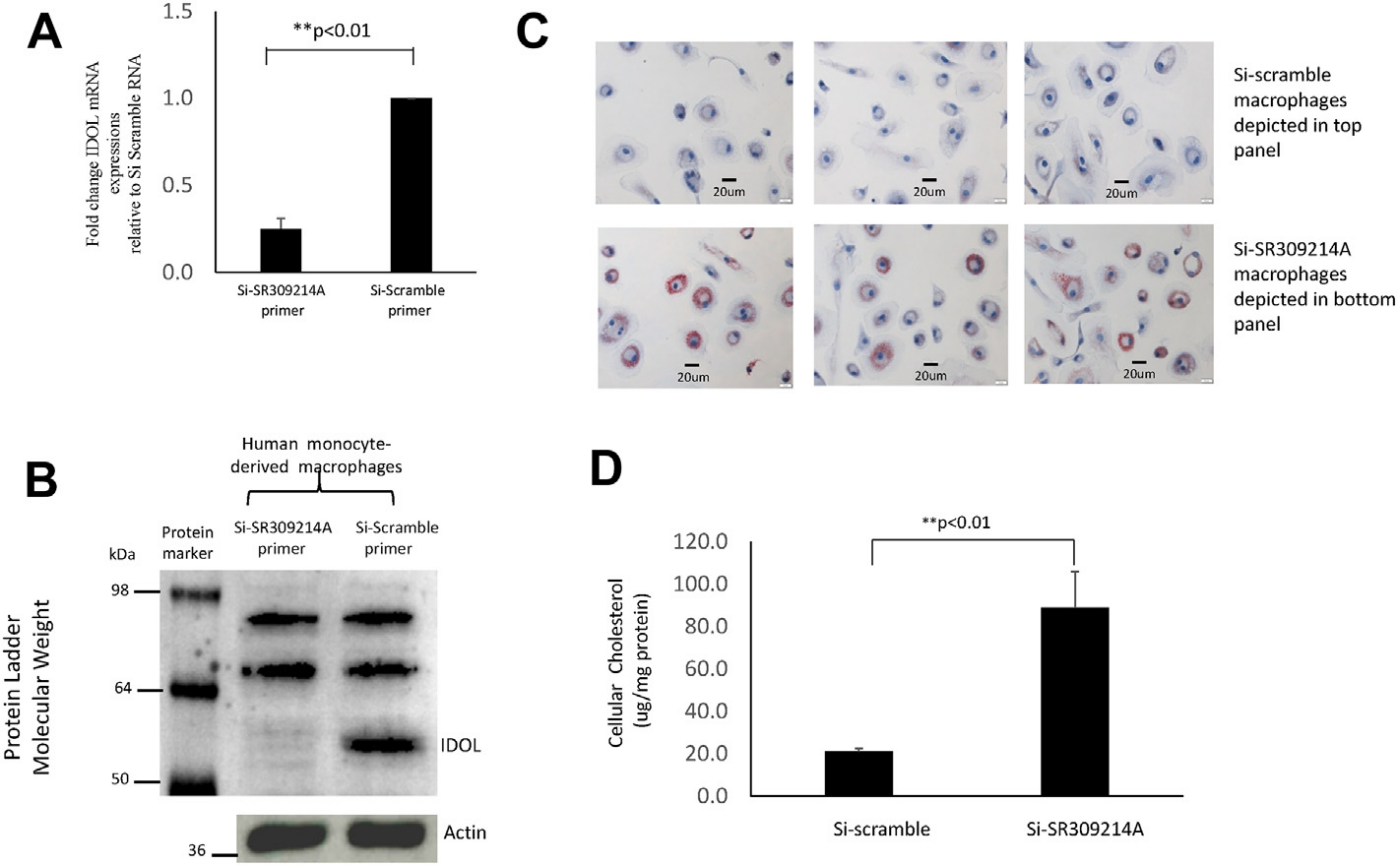
Human monocyte-derived macrophages from four healthy subjects were transfected with Si-SR309214A or Si-scramble control SiRNA, and quantitative mRNA levels (A) and Western blot (B) confirmed the efficiency of IDOL knockdown 72 h after transfection. Macrophages incubated with 10 μg/ml LDL for 6 h and stained with Oil Red O to visualize lipid accumulation showed an increase in intracellular lipid accumulation in IDOL knockdown macrophages (C) and an increase in cellular cholesterol levels (D).

**Fig. 6:**
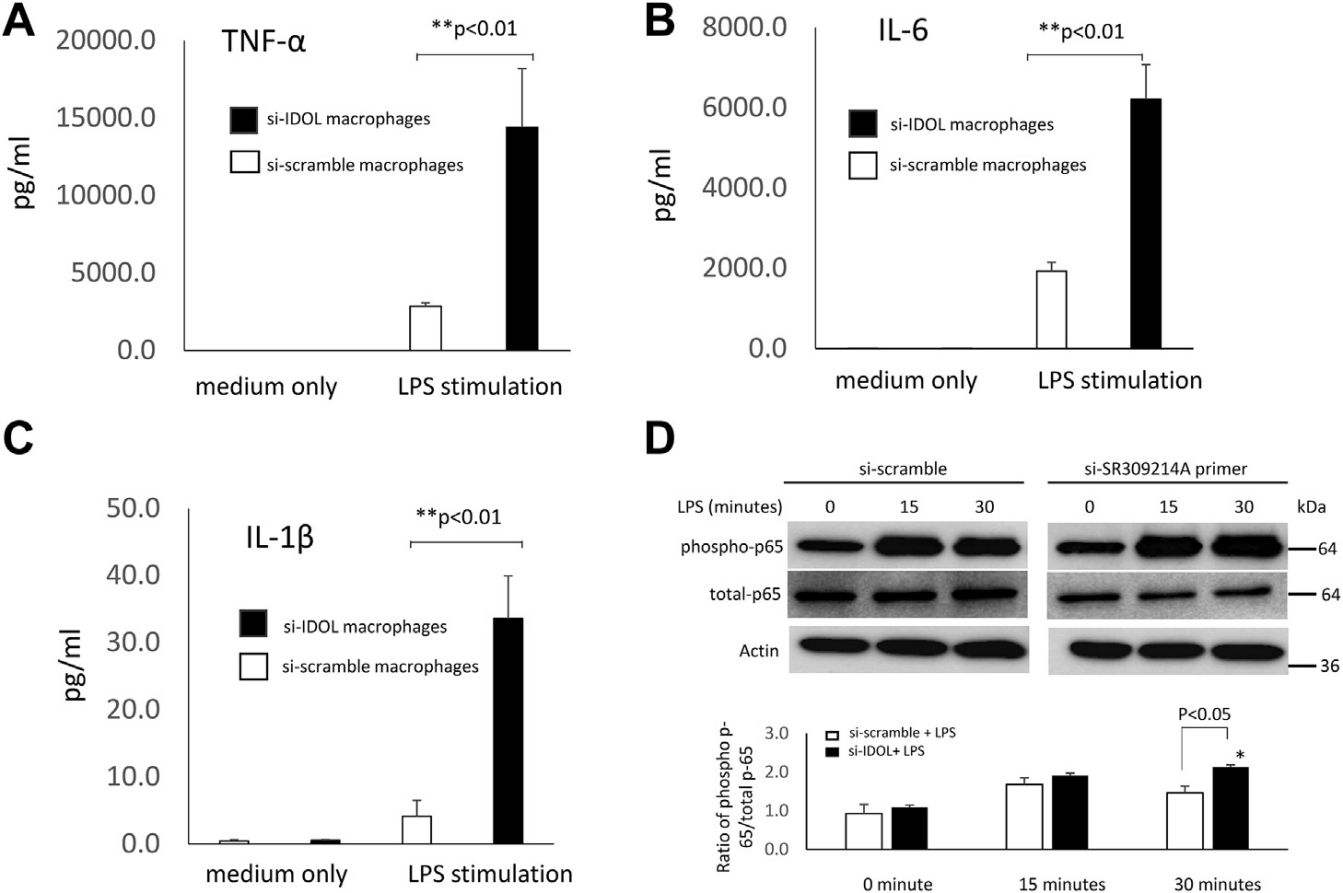
Cytokine production of TNFα (A), IL-6 (B), IL-1β (C), and activation of NF-kB p65 (D) in IDOL knockdown and control macrophages. Data represent the mean ± SD of four experiments. ∗*P* < 0.05, ∗∗*P* < 0.01 versus Si-scramble control macrophages. The statistical significance of differences was determined using Student’s *t*-test.

RNA silencing experiments were performed to verify that IDOL knockdown in human monocyte-derived macrophages resulted in increased lipid accumulation and to further investigate whether changes in IDOL expression affected the function of macrophages. Knockdown of IDOL resulted in a significant reduction in IDOL expression (Fig. 5A, B). Macrophages were stained with Oil Red O to visualize lipid accumulation, and there was an increase in intracellular lipid accumulation in IDOL knockdown macrophages (Fig. 5C). There were no significant differences in inflammatory cytokine production between IDOL knockdown macrophages and control macrophages without stimulation. However, IDOL knockdown macrophages produced much higher concentrations of TNFα, interleukin 6, and interleukin 1 beta than control macrophages when LPS was added (Fig. 6), and the hyperinflammatory response in IDOL knockdown macrophages was partly because of increased NF-kB activation (Fig. 6D).

## Discussion

IDOL is a post-transcriptional degrader of LDLR. Recent studies have shown that IDOL is active not only in the liver but also in peripheral tissues and may have a physiological role beyond systemic cholesterol homeostasis (11, 12, 13, 14). For instance, IDOL is highly expressed in the hippocampus, and loss of IDOL impairs plasticity and memory formation in animal studies (14). It is unclear how IDOL is transcriptionally regulated in different cell types and tissues. We have specifically investigated the role and regulation of IDOL in macrophages, as IDOL is the only post-transcriptional degrader of LDLR in macrophages and act intracellularly by ubiquitination of the intracellular tail of LDLR. We have measured the expression of IDOL in CD14+ monocytes in subjects with and without type 2 diabetes and shown that IDOL expression in CD14+ monocytes was significantly reduced in individuals with diabetes compared with healthy nondiabetic control. The expression of IDOL in CD14+ monocytes was associated with glycemia and serum FGF21 level. Our results concur with those reported by Ding *et al.* (20) who investigated the relationships between BMI and the transcriptional signature in monocytes in participants from the Multi-Ethnic Study of Atherosclerosis. In their study, *IDOL/MYLIP* was one of the genes that were downregulated in monocytes with increasing BMI. Although we did not find an association between IDOL expression and BMI, which might be due to differences in the population being studied, the reduction of IDOL expression in obese individuals and in those with type 2 diabetes might both be related to serum FGF21 levels. Circulating FGF21 concentration is known to be increased in obesity and type 2 diabetes (22, 26, 27). In our study, FGF21 level was independently associated with IDOL expression, and FGF21 reduced IDOL expression in a dose-dependent manner in vitro. How IDOL is regulated by FGF21 has been elucidated by Do *et al.* (21) who reported that FGF21 reduced IDOL expression by increasing the expression of Canopy2/MIR-interacting saposin-like protein, an inhibitor of IDOL, in macrophages and hepatocytes in vitro. In addition to FGF21, we have shown that glycemia may also play an important role in the regulation of IDOL expression as HbA1c was a major determinant of IDOL expression in CD14+ monocytes. Experimental studies have shown that glucose concentration can modulate the expression of LXR-dependent genes like *ABCA1* in macrophages (19), and we have shown that glucose can also directly downregulate IDOL in vitro.

What is the effect of reducing IDOL expression in CD14+ monocytes? As expected, the reduction of IDOL expression in CD14+ monocytes was accompanied by an increase in LDLR. Using labeled LDL particles, we demonstrated that LDL binding and intracellular lipid accumulation was also increased. Knockdown of IDOL in human monocyte-derived macrophages also resulted in lipid accumulation. Intracellular lipid accumulation in macrophages is a net result of native as well as modified LDL uptake by LDLR and scavenger receptors, respectively, and the efflux of cholesterol by cholesterol transporters (28, 29). We and others have previously shown that the expression of ABCG1 in monocytes was reduced in patients with type 2 diabetes (24, 25), and increased intracellular lipid accumulation has been attributed to the decrease in ABCG1 expression and cholesterol efflux (24). We have now shown that the reduction in IDOL expression also contributed to the increase in intracellular lipid accumulation independent of ABCG1 in patients with type 2 diabetes because the inverse correlation between IDOL expression and intracellular lipid accumulation remained significant even after controlling for ABCG1 expression.

Whether the reduction in IDOL expression and the concomitant increase in intracellular lipid accumulation in CD14+ monocytes in type 2 diabetes has any adverse consequences is unclear. Monocytes are important cells of innate immunity and contribute to the pathogenesis of atherosclerosis. Macrophages in atherosclerotic plaques are derived from circulating monocytes and local proliferating plaque macrophages. Lipoprotein uptake and lipid accumulation by arterial macrophages giving rise to foam cells is a key process in the formation of atherosclerotic plaques. High fat meals can induce lipid loading in circulating monocytes before their migration into tissues and differentiate into macrophages (30). Hence, reducing IDOL expression in monocytes/macrophages may conceivably contribute to foam cell formation. Moreover, the extent of intracellular lipid loading can significantly affect macrophage functions (31). Macrophages are known to participate in the inflammatory process in atherosclerosis (28, 29, 31). Activated macrophages contribute to the local inflammatory response in atherosclerotic plaques and secrete proinflammatory cytokines and chemokines. We have performed knockdown experiments to evaluate the effect of modulating IDOL expression on the inflammatory response of macrophages. We observed a significant increase in the production of proinflammatory cytokines upon stimulation with LPS, a Toll-like receptor 4 ligand. Hence, macrophages with reduced IDOL expression appear to display a more proinflammatory phenotype when stimulated. This is similar to the observation that monocytes in patients with symptomatic atherosclerosis have a proinflammatory phenotype. Bekkering *et al.* (32) have shown that circulating monocytes from these individuals produced significantly more proinflammatory cytokines upon LPS stimulation ex vivo than healthy controls. Our preliminary data suggest that the enhanced LPS-induced cytokine expression in IDOL knockdown cells is partly because of increased activation of NF-kB. Whether the enhanced inflammatory response is also related to altered cellular cholesterol content and/or activation of mitogen-activated protein kinase pathways remains to be determined. The specific role of IDOL in macrophages in the pathogenesis of atherosclerosis and the mechanism(s) involved in the modulation of IDOL expression on inflammatory response therefore warrants further investigation.

Our study has some limitations. Type 2 diabetic cohort was significantly older than the control cohort. Causality of the relationship between glycemia, FGF21, and IDOL cannot be determined because of the cross-sectional design of our study. We also cannot address the association of IDOL in CD14+ monocytes with atherosclerosis. None of our recruited subjects had cardiovascular disease because statin therapy was one of the exclusion criteria. Although the reduction of IDOL was associated with increased LDLR expression and intracellular lipid accumulation in individuals with diabetes, the relative contribution of IDOL and LDLR-mediated lipoprotein uptake to intracellular lipid accumulation in macrophages still needs to be further evaluated since we have not measured scavenger receptor expression and modified lipoprotein uptake in our study. Furthermore, we cannot rule out the possibility that the differences in intracellular lipid accumulation in CD14+ monocytes seen in vivo may be a reflection of the dyslipidemia present in subjects with type 2 diabetes. Finally, the changes in IDOL expression observed in monocyte samples may not reflect changes in other tissues or cells.

In conclusion, the expression of IDOL in CD14+ monocytes was decreased in subjects with type 2 diabetes and was associated with glycemia and serum FGF21 concentration. The reduction in IDOL expression was accompanied by an increase in intracellular lipid accumulation.

## Data availability

The summary data that support the findings of this study are available from the corresponding author upon reasonable request.

## Supplemental data

This article contains supplemental data.

## Conflict of interest

The authors declare that they have no conflicts of interest with the contents of this article.
